# Animal contact and paediatric acute febrile illness in Greater Accra Region, Ghana

**DOI:** 10.4314/gmj.v56i3.13

**Published:** 2022-09

**Authors:** Melissa N Sidote, Justin Stoler, Nicholas Amoako, Samuel Duodu, Gordon Awandare

**Affiliations:** 1 Department of Public Health Sciences, Miller School of Medicine, University of Miami, Miami, FL, USA; 2 Department of Geography and Sustainable Development, University of Miami, Coral Gables, FL, USA; 3 Kintampo Health Research Centre, Kintampo, Ghana; 4 West African Centre for Cell Biology of Infectious Pathogens, University of Ghana, Accra, Ghana; 5 Department of Biochemistry, Cell and Molecular Biology, University of Ghana, Legon, Ghana

**Keywords:** fever, acute febrile illness, bacterial zoonosis, pet infections

## Abstract

**Objective:**

To examine the association between animal contact (primarily dogs and cats) and non-malarial fever, as well as with secondary symptoms of headache, nausea, vomiting, and cough, in 687 children in Greater Accra Region, Ghana.

**Design:**

Cross-sectional study of acute febrile illness among children aged 1–15 years old between October 2016 and August 2017.

**Setting:**

Ledzokuku-Krowor Municipal Assembly (LEKMA) Hospital, Teshie, Greater Accra Region.

**Participants:**

The study included children with acute fever, defined as a measured temperature of greater than 37.5°C, occurring less than seven days before the hospital visit, and afebrile children as controls.

**Main outcome measures:**

Measured fever, self-reported fever, and secondary symptoms, each adjusting for patient household characteristics.

**Results:**

Animal contact was neither associated with measured fever (OR = 1.04, 95% CI 0.73–1.49) nor with self-reported fever (OR = 0.97, 95% CI 0.68–1.39). Animal contact was associated with headache (OR = 3.26, 95% CI 2.23–4.77, *P* < .01) and nausea (OR = 3.05, 95% CI 1.99–4.68, *P* < .01), but not with vomiting or cough. Additional models that used alternate inclusion criteria to define non-malarial fever yielded similar results. Several bacterial zoonoses that could plausibly have been transmitted by dogs and cats were diagnosed in the study population.

**Conclusion:**

These findings suggest the need for future studies to evaluate animal contact as a risk factor for bacterial zoonoses that may serve as an etiological driver of acute febrile illness.

**Funding:**

no external funding

## Introduction

Acute febrile illness, a rapid onset of fever occurring for less than 7 days, causes a large burden of disease globally.^1^,^2^ Febrile illness is among the most common reasons for people in low-resource areas to seek healthcare.^3^,^4^ In sub-Saharan Africa, fever is a common symptom of many important sources of morbidity and mortality.^2^ Annually, over six million children die due to preventable or treatable illnesses, many of which are febrile, and approximately 50% are in resource-limited settings.^5^

Undifferentiated fever is the main clinical symptom of many diseases of global importance, including malaria, bacterial diseases, bacterial zoonoses and many viral infections.^3^ But the diagnosis of patients with febrile illness is challenging due to both a lack of specific presentation and limited diagnostics.^2^,^6^

Except for malaria diagnostics, laboratory tests for many febrile diseases are often complex, costly, and not widely available in low-resource areas.^1^,^3^ While malaria remains a major cause of fever in tropical sub-Saharan Africa, the incidence of malaria has been declining since 2003,^2^ and recent studies have demonstrated that a significant proportion of febrile patients suspected of malaria are actually suffering from other infections.^2^,^4^

Efforts to better understand the causes of febrile illness have identified bacterial zoonoses as common, under-recognized causes of fever in Africa.^2^,^4^ In a study of 870 febrile children and adults in Northern Tanzania, bacterial zoonoses were identified among 26.2% of clinic admissions.^4^ Household animals, such as dogs and cats, are known to transmit bacterial zoonoses such as leptospirosis, brucellosis, and Q fever, as well as common infections such as *staphylococci*, rickettsia, and *Streptococcus pneumoniae.*^2^,^7^–^10^

Both pets and feral animals can become vectors of bacterial zoonoses in settings where inadequate water, sanitation, and hygiene (WASH) services expose free-ranging animals to waste and pathogens in the environment. Exposed animals may then carry pathogens into human living spaces and create subsequent infection pathways similar to faecal-oral contamination from food, water, and fomites,^11^ and physical contact between humans and outdoor-living animals. Children's outdoor play spaces may present additional transmission pathways if they overlap with animal activity spaces. These types of animal exposures are typical of the urban ecology of low-income communities with insufficient WASH services^12^, particularly concerning rodents^13^, but a few studies have examined the potential of such public health burden in the African context associated with dogs and cats.

Understanding the full range of aetiologies of acute febrile illness has important implications for prevention and treatment. This study examined the association between animal contact (primarily dogs and cats) and fever, as well as secondary symptoms of headache, nausea, vomiting, and cough, in paediatric acute febrile illness patients in Teshie, a large town in the Greater Accra Region, Ghana. Our goal was to assess whether animal contact might be a risk factor for febrile illness in this setting and whether bacterial and other zoonoses warrant additional exploration as a source of undifferentiated fever.

## Methods

Children aged 1–15 years old at the outpatient clinics of the Ledzokuku-Krowor Municipal Assembly (LEKMA) Hospital in the Greater Accra Region in southern Ghana were recruited to a cross-sectional study of acute febrile illness between October 2016 and August 2017. LEKMA was a coastal district between the Accra Metropolitan Area and Tema with a 2010 population of 227,932^14^ and anchored by two large towns, Teshie and Nungua (it was split into two municipal districts in 2018). This region has experienced recent economic development, but despite most households having piped water connections, most of the district still lacks reliable WASH services in many areas due to water rationing.^15^ Household animal ownership—which is typically dogs and cats, but occasionally goats, fowl, or cattle—is similar to the rates observed in other Ga communities in Accra, such as Bukom and Shiabu.^11^

The study included children with acute fever, defined as a measured temperature of greater than 37.5°C, occurring less than seven days before the hospital visit, and afebrile children as controls. The clinician also recorded whether the fever was self-reported by the child or parent/guardian. Additional inclusion criteria were the parent or guardian's willingness to provide informed consent, with the willingness of the child if over 10 years old, residency in the study area for two months, and parent/guardian consent to allow blood and other samples to be collected. Children showing signs of severe disease, children with a chronic illness, and minors unaccompanied by a parent or guardian were excluded from the study.

Because malaria is not transmitted via household animals, we excluded children diagnosed with malaria from our analysis of animal contact. For robustness, we tested two separate criteria of “malaria-negative”: children with a negative parasite test or malaria RDT, and those who did not receive a clinical diagnosis of malaria from the attending physician. As already noted, fever also had two possible definitions: fever as self-reported by participants and temperature measured at the clinic. To ensure that our results were stable across clinical definitions of malaria and fever, we repeated our analysis of animal contact for all four combinations of inclusion criteria and fever diagnosis, from most to least objective: (1) parasite negative (for malaria), measured fever; (2) parasite negative, self-reported fever; (3) clinical negative (for malaria), measured fever; (4) clinical negative, self-reported fever. This robustness was important given how, in some regions of Ghana, traditional words for *fever* and *malaria* have historically been interchangeable.^16^

**Figure S1:**
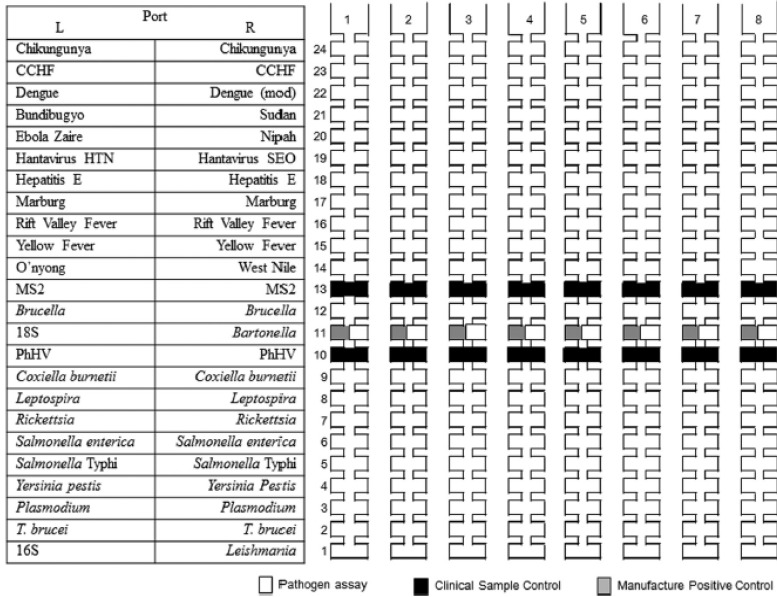
Configuration of TaqMan array card with list of pathogens that were tested in the parent febrile illness study.

The study team, comprised of a field worker and a nurse, identified children and measured their temperature, height and weight. The study information and procedures were explained to every parent or guardian with sufficient time to contemplate participation. The study team obtained written informed consent from all parents/guardians. The participants were then sent to the study clinician who physically examined them and enrolled them in the study. A questionnaire was administered to collect socio-demographic and additional clinical data, including signs and symptoms. It included an item assessing whether the child had contact with animals (alive or dead) in the prior two weeks. All children were tested for malaria using microscopy and rapid diagnostic test (RDT). Blood samples from children with negative malaria RDT and microscopy were randomly selected by using a random number generator to select sample IDs to be further analysed using a customised multi-pathogen, real-time PCR-based TaqMan probe-array card (TAC; Applied Biosystems, Carlsbad, CA, USA), as described else-where.^17^ The TaqMan Array Card assessed 26 pathogens, including several bacterial zoonoses (Figure S1, Supplementary Material).

Additional blood and urine cultures were performed at the request of the clinician. Nasopharyngeal samples were collected from participants showing signs of respiratory infection; however, laboratory analyses of these samples were not completed due to resource constraints.

The study team performed bivariate analyses of participant demographic characteristics using chi-square tests for categorical variables and *F* tests for the difference in means between groups for continuous variables. We fitted multivariable logistic regression models for each of the four combinations of fever and malaria definitions to analyse the association between animal contact and fever, adjusting for covariates. We controlled for several participant characteristics, including gender, parent education, parent occupation, ethnicity, and whether a drug was being taken for the current illness at the time of the visit. We then used chi-square tests to explore bivariate associations between the two definitions of fever and the most common secondary symptoms potentially related to animal-transmitted diseases (headache, nausea, vomiting and cough). We then performed a similar multivariable regression analysis of these symptoms. All analyses were performed using the software R version 3.6.2.

### Ethical Approval

The original febrile illness study was approved by the Institutional Review Committee of Kintampo Health Research Centre (approval number: 0004854), Institutional Review Board of Noguchi Memorial Institute for Medical Research at the University of Ghana (study number: 099/15-16), and Ghana Health Service Ethical Review Committee (GHS-ERC number: 12/06/2016). Informed consent was sought from the caregivers of the participants. Confidentiality and anonymity were ensured throughout the process. This study, conducted on anonymised patient data, was deemed non-human subjects research by the Institutional Review Board at the University of Miami.

## Results

Our analytical sample sizes were reduced from 775 to 687 after excluding 88 children due to parasite-positive malaria, and from 775 to 593 after excluding 182 children receiving a clinical malaria diagnosis. Table 1 summarises patient characteristics according to each definition of fever; 59% self-reported fever, and 54% had a measured fever at the clinic.

**Table 1:** Descriptive characteristics of select patient and household characteristics showing associations with measured and self-reported fever (*n* = 687)

	Measured			
	Febrile	Afebrile	Febrile	Afebrile
**Overall Rate**	368 (54%)	319 (46%)	406 (59%)	281 (41%)
**Age (years)**	3.45 (2.83)***	5.42 (4.23)	3.47 (2.78)***	5.75 ( 4.33)
**Gender**				
**Female**	169 (46%)	164 (51%)	189 (47%)	144 (51%)
**Male**	199 (54%)	155 (49%)	217 (53%)	137 (49%)
**Yes**	103 (28%)	86 (27%)	111 (27%)	78 (28%)
**No**	265 (72%)	233 (73%)	295 (73%)	203 (72%)
**Cat**	58 (16%)	53 (17%)	64 (16%)	47 (17%)
**Cat & Dog**	1 (<1%)	0 (0%)	1 (<1%)	0 (0%)
**Dog**	41 (11%)	32 (10%)	43 (11%)	30 (11%)
**Sheep/Goat**	2 (<1%)	1 (<1%)	2 (<1%)	1 (<1%)
**Unknown**	1 (<1%)	0 (0%)	1 (<1%)	0 (0%)
**None**	24 (7%)	26 (8%)	26 (6%)	24 (9%)
**Primary**	137 (37%)	109 (34%)	151(37%)	95 (33%)
**Secondary**	122 (33%)	111 (35%)	136 (33%)	97 (34%)
**Tertiary**	85 (23%)	73 (23%)	93 (23%)	65 (23%)
**Professional, skilled labour**	92(25%)***	116 (36%)	106 (26%)***	102 (36%)
**Trader, unskilled labour,** **unemployed**	276 (75%)	203 (64%)	300 (74%)	179 (64%)
**Ethnicity**				
**Ga Adangbe**	143 (39%)	103 (32%)	153 (38%)	93 (33%)
**Akan**	119 (32%)	118 (37%)	135 (33%)	102 (36%)
**Ewe**	71 (19%)	75 (24%)	81 (20%)	65 (23%)
**Northern**	27 (7%)	19 (6%)	29 (7%)	17 (6%)
**Unknown**	8 (2%)	4 (1%)	8 (2%)	4 (1%)
**None**	104(28%)***	160 (50%)	114 (28%)***	150 (53%)
**Analgesic**	165 (45%)	113 (35%)	188 (46%)	90 (32%)
**Other**	99 (27%)	46 (14%)	104 (26%)	41 (15%)
**Yes**	13 (4%)	0 (0%)	13 (3%)	0 (0%)
**No**	354 (96%)	318 (100%)	391 (96%)	281 (100%)
**Pipe borne water**	12 (3%)	1 (<1%)	12 (3%)	1 (0.3%)
**Sachet water**	356 (97%)	318 (99%)	394 (97%)	280 (99%)

* *P* < .05, ** *P* < .01

*** *P* < .001

^†^ Associations not measured due to lack of variation

Regardless of fever definition, parasite-negative, febrile children were significantly younger than afebrile children, more likely to have parents employed in lower-skilled jobs or unemployed, and more likely to have been taking a drug for their illness at the time of visit (all *P* < .001) (Table 1). We observed the same differences between febrile and afebrile children using the alternate inclusion criteria of being clinically diagnosed as negative for malaria (Table S1, Supplementary Material). Among parasite-negative children, 28% of febrile (measured) and 27% of afebrile children reported contact with animals 14 days prior to the clinic visit, with similar rates observed when defining fever as self-reported. Animal exposures, defined as contact with any animal (alive or dead) in the past two weeks, were most commonly cats and dogs, with two febrile children reporting contact with sheep or goats.

Table S1 (see Supplementary Material) summarizes the characteristics of patients based on the secondary inclusion criteria of no malaria clinical diagnosis. A total of 593 patients were included in this analysis, 59% of which reported fever and 54% had a measured fever at the clinic. Ninety-three children, or 27% of febrile children, and 88 children, or 28% of children with a measured fever, reported contact with animals in the 14 days prior to their clinic visit.

Table 2 summarises the four multivariable logistic regression models for each inclusion criterion. Among children with a parasite-negative malaria test, animal contact was neither associated with self-reported fever (OR = 0.97, 95% CI 0.68–1.39), nor with a measured fever (OR = 1.04, 95% CI 0.73 -1.49), with similar results for children diagnosed as clinically negative for malaria.

**Table 2:** Multivariable logistic regression models of the association between patient characteristics and fever, using two fever criteria (measured vs. self-reported) and two methods of malaria diagnosis (parasite-negative vs. clinical negative)

	Measured Fever & Parasite Negative	Self-Reported Fever & Parasite Negative	Measured Fever & Clinical Negative	Self-Reported Fever & Clinical Negative
**Animal Contact**				
**No** †	--	--	--	--
**Yes**	1.04 (0.73–1.49)	0.97 (0.68–1.39)	0.99 (0.68–1.44)	0.89 (0.61–1.30)
**Gender**				
**Male** †	--	--	--	--
**Female**	0.79 (0.58–1.08)	0.82 (0.59–1.13)	0.79 (0.56–1.10)	0.80 (0.57–1.13)
**Parent Education**				
**None** †	--	--	--	--
**Primary**	1.64 (0.86–3.17)	1.72 (0.89–3.32)	1.17 (0.59–2.33)	1.39 (0.69–2.76)
**Secondary**	1.28 (0.67–2.46)	1.36 (0.70–2.61)	0.98 (0.49–1.95)	1.21 (0.61–2.41)
**Tertiary**	1.54 (0.77–3.13)	1.51 (0.74–3.06)	1.18 (0.56–2.49)	1.37 (0.65–2.87)
**Parent Occupation**				
**Trader, unskilled labour,** **unemployed** †	--	--	--	--
**Professional/skilled labour**	0.53 (0.36–0.75)***	0.56 (0.39–0.83)**	0.54 (0.36–0.79)**	0.60 (0.41–0.89)*
**Ethnicity**				
**Ga Adangbe** †	--	--	--	--
**Akan**	0.75 (0.52–1.09)	0.83 (0.57–1.22)	0.76 (0.51–1.14)	0.80 (0.53–1.21)
**Ewe**	0.75 (0.48–1.15)	0.83 (0.53–1.28)	0.74 (0.47–1.16)	0.78 (0.49–1.24)
**Northern**	1.04 (0.53–2.06)	1.01 (0.51–2.05)	1.12 (0.53–2.39)	0.91 (0.44–1.95)
**Unknown**	1.31 (0.39–5.15)	1.05 (0.31–4.14)	1.19 (0.34–4.79)	0.96 (0.27–3.83)
**Drug for current illness**				
**None** †	--	--	--	--
**Analgesic**	2.19 (1.54–3.12)***	2.71 (1.90–3.89)***	2.07 (1.42–3.03)***	2.36 (1.62–3.47)***
**Other**	3.48 (2.25–5.45)***	3.52 (2.26–5.54)***	3.38 (2.12–5.46)***	3.15 (1.97–5.09)***

Odds ratios (OR) presented with 95% confidence intervals (95% CI).

* *P* < .05

** *P* < .01

*** *P* < .001

† Reference category

Having a parent's occupation categorised as professional/skilled labour was significantly associated with measured fever (OR = 0.53, 95% CI 0.36–0.75), as well as taking either an analgesic drug (OR = 2.19, 95% CI 1.54–3.12) or other types of drug (OR = 3.48, 95% CI 2.25–5.45) for the current illness. We observed similar results for all four multivariable models (Table 2).

Across the four columns of Table 2, which represent decreasing objectivity of diagnosis from left to right, two of the three significant measures—parent occupation and using ‘other’ drug as treatment—had the smallest effects in the least objective combination of self-reported fever and being clinically negative for malaria.

Although the differences were modest, this underscores the reason for testing the four different combinations of fever and malaria-negative definitions, given how diagnostic capacity can vary throughout the country.

Table 3 summarises associations between measured fever and reported secondary symptoms. Among the 687 patients with a negative parasite test or RDT, 115 patients reported nausea, 171 headache, 196 vomiting and 274 cough. Measured fever was significantly associated with headache, χ^2^(1, N = 687) = 8.85, *P* < .01 and cough, χ^2^(1, N = 687) = 5.48, *P* = .02, while self-reported fever was significantly associated with headache, χ^2^(1, N = 687) = 5.29, *P* = .02 and vomiting, χ^2^(1, N = 687) = 4.74, p = .04. Nausea was significantly associated with headache, χ^2^(1, N = 687) = 51.22, *P* < .001, and vomiting, χ^2^(1, N = 687) = 213.91, *P* < .001. Vomiting was significantly associated with cough, χ^2^(1, N = 687) = 13.54, *P* < .001.

**Table 3:** Chi-square matrix showing associations between symptoms

	Measured Fever	Self-Reported Fever	Headache	Nausea	Vomiting	Cough
**Measured Fever**		512.20***	8.85**	0.65	1.45	5.48*
**Self-Reported Fever**	512.20***		5.29*	0.65	4.74*	2.23
**Headache**	8.85**	5.29*		51.22***	0.20	2.76
**Nausea**	0.65	0.65	51.22***		213.91***	0.02
**Vomiting**	1.45	4.74*	0.20	213.91***		13.54***
**Cough**	5.48*	2.23	2.76	0.02	13.54***	

* *P* < .05

** *P* < .01

*** *P* < .001

Table 4 summarises the multivariable logistic regression models of headache, nausea, vomiting, and cough with animal contact, again controlling for covariates. There was an association between animal contact and headache for patients with a parasite-negative test result (OR = 3.26, 95% CI 2.23–4.77, *P* < .01) and for patients without a clinical malaria diagnosis (OR = 2.95, 95% CI 1.96–4.42, *P* < .01). Animal contact was also associated with nausea for patients with a parasite negative test results (OR = 3.05, 95% CI 1.99–4.68, *P* < .01) and for patients without a clinical diagnosis of malaria (OR = 2.72, 95% CI 1.71–4.32, *P* < .01). Animal contact was not associated with vomiting or cough.

**Table 4:** Multivariable logistic regression models of the association between patient characteristics and headache, nausea, vomiting and cough, using two inclusion criteria

	Headache (Parasite Negative)	Headache (Clinical Negative)	Nausea (Parasite Negative)	Nausea (Clinical Negative)	Vomit (Parasite Negative)	Vomit (Clinical Negative	Cough (Parasite Negative)	Cough (Clinical Negative)
**Animal Contact**								
**No** †	--	--	--	--	--	--	--	--
**Yes**	3.26 (2.23–4.77)***	2.95(1.96–4.42)***	3.05 (1.99–4.68)***	2.72 (1.71–4.32)***	0.97(0.66–1.41)	0.88(0.581.32)	0.86 (0.60–1.21)	0.82(0.56–1.19)
**Gender**								
**Male** †	--	--	--	--	--	--	--	--
**Female**	1.09 (0.76–1.58)	1.09 (0.74–1.61)	1.04 (0.68–1.59)	1.09 (0.69–1.71)	1.27(0.90–1.78)	1.18(0.82–1.69)	1.08 (0.79–1.47)	1.17 (0.84–1.63)
**Parent Education**								
**None** †	--	--	--	--	--	--	--	--
**Primary**	0.65 (0.33–1.36)	0.76 (0.36–1.67)	0.54 (0.23–1.18)	0.54 (0.25–1.23)	1.21(0.62–2.47)	0.97 (0.48–2.02)	1.02 (0.54–1.93)	1.07 (0.55–2.10)
**Secondary**	0.59 (0.29–1.23)	0.73 (0.35–1.61)	0.39 (0.18–0.87)*	0.38(0.170.89)*	0.79(0.40–1.64)	0.75 (0.37–1.57)	0.76 (0.41–1.45)	0.80 (0.41–1.58)
Tertiary	0.75 (0.35–1.64)	1.00 (0.45–2.31)	0.57 (0.25–1.33)	0.57 (0.24–1.38)	0.94(0.45–2.01)	0.81 (0.38–1.79)	0.71 (0.36–1.42)	0.74 (0.36–1.54)
**Parent Occupation**								
**Professional,** **skilled labour**	1.16 (0.77–1.75)	1.13 (0.73–1.75)	0.93 (0.57–1.48)	0.78 (0.46–1.32)	1.16(0.79–1.69)	1.08 (0.71–1.62)	1.13 (0.79–1.61)	1.12 (0.76–1.65)
**Ethnicity**								
**Ga Adangbe** †	--	--	--	--	--	--	--	--
**Akan**	1.22 (0.79–1.88)	1.31 (0.83–2.08)	0.93 (0.56–1.52)	0.92 (0.54–1.58)	1.09(0.73–1.64)	1.09(0.711.68)	1.34(0.93–1.95)	1.28 (0.86–1.91)
**Ewe**	0.99 (0.59–1.65)	1.05 (0.61–1.78)	0.98 (0.55–1.71)	1.05 (0.57–1.91)	0.99(0.62–1.58)	1.04(0.631.69)	1.04 (0.68–1.59)	1.06 (0.67–1.68)
**Northern**	1.00 (0.44–2.16)	1.18 (0.49–2.68)	0.57 (0.18–1.49)	0.73 (0.23–1.94)	1.34(0.65–2.64)	1.19(0.542.52)	1.07 (0.54–2.05)	0.99 (0.47–2.04)
**Unknown**	1.56 (0.29–6.37)	1.77 (0.34–7.51)	0.43 (0.02–2.69)	0.46 (0.02–3.06)	0.59(0.09–2.38)	0.27(0.011.52)	1.29 (0.37–4.21)	1.39 (0.38–4.84)
**Drug for current illness**								
**None** †	--	--	--	--	--	--	--	--
**Analgesic**	0.96 (0.63–1.46)	0.94 (0.59–1.47)	0.57 (0.34–0.94)*	0.57(0.320.98)*	1.03(0.69–1.52)	1.03(0.681.58)	1.41 (0.99–2.01)	1.43 (0.98–2.11)
**Other**	1.70 (1.05–2.73)*	1.63 (0.98–2.71)	1.56 (0.93–2.60)	1.93(1.113.34)*	1.89(1.212.94*	1.90(1.183.07*	2.02 (1.33–3.09)**	2.17 (1.38–3.42)***

Odds ratios (OR) presented with 95% confidence intervals (95% CI).

* *P* < .05

** *P* < .01

*** *P* < .001

† Reference category

Blood and urine tests yielded positive culture results for just seven febrile children with a parasite-negative test who reported animal contact (see Table 5). Three children had coagulase-negative *staphylococci*, and there was one case of *Enterococcus* spp, HIV, *Staphylococcus aureus*, and *Streptococcus pneumoniae*. Additionally, among children who did not report animal contact, test results included dengue virus, *Escherichia coli*, group D streptococci, *Pseudomonas aeruginosa*, Rickettsia, *Staphylococcus aureus*, and *Streptococcus pneumoniae*, underscoring the range of infections identified in the original parent study.^18^,^19^

## Discussion

This study found that animal contact was neither associated with self-reported nor measured fever in malaria-negative children. Animal contact was associated with the secondary symptoms of headache and nausea, common yet non-specific symptoms of possible animal-transmitted infections, including leptospirosis, Q fever, and Campylobacteriosis.20,21 These findings, although inconclusive, may be attributed to the vast aetiology of fever, one or more undiagnosed zoonoses that present with headache and nausea but without fever, or the lack of context around the type of animal contact reported. But these associations beckon further study of the potential burden of bacterial zoonoses on undifferentiated paediatric fevers.

In the context of rising zoonosis infections globally—particularly in the wake of the COVID-19 pandemic—Ghana may still benefit from a richer understanding of the prevalence and common symptoms of endemic zoonoses. Our results demonstrating borderline weaker associations between social determinants of health and the least objective forms of diagnosis (self-reported fever and clinical malaria) suggest that diagnostic technology continues to reproduce disparities in treatment.

While this study focused primarily on contact with dogs and cats, future studies might also consider contact with livestock such as goats and cattle, particularly in peri-urban and urban environments where higher population density can facilitate transmission, with a more detailed characterisation of animal contact, and a broader, more specific set of symptoms.

Household animals are increasingly recognised as reservoirs for neglected infections. For example, it was originally thought that ownership of cats or dogs did not increase the risk of Rickettsia,^22^ but more recent studies have found pets to be an emerging reservoir after all.^8^,^23^–^25^ Dog ownership has been established as a risk factor for Campylobacter, recognised as one of the most common bacterial infections associated with pet ownership.^21^,^26^,^27^ Coagulase-negative *staphylococci* have been found in clinically healthy dogs,^28^ and have the ability to transfer between pets and their owners, contributing to an increased risk of *staphylococci* infections.^7^ A case of identical *Staphylococcus intermedius* strains found in a woman and her dog highlighted the risk of transmission of bacterial infections from household pets to humans.^29^ The infections detected in the present study, except HIV, could plausibly have been transmitted by animal contact, among diverse potential origins.^7^,^9^,^30^,^31^ The HIV case was a ten-year-old boy, and we retained this case in the analysis because it was plausible that the observed symptoms were due to some other undetected infection, not HIV. This suggests that more research is needed to evaluate animal contact as a risk for bacterial zoonoses, which may serve as an etiological driver of acute febrile illness in sub-Saharan Africa.

This study was limited by its cross-sectional design and limited data resolution about the patients'-built environment, outdoor activities, and nature or frequency of animal contact. It is plausible that feral animals pose different risks than pets or that specific transmission pathways shape outcomes more than the mere presence of animals. Additionally, the household data were self-reported by the child's guardian during recruitment at the clinic, which may contribute to selection and recall biases. The lack of available blood tests and nasopharyngeal results for every child meant that a majority of the fever cases remained unidentified. In a related study, efforts to model paediatric febrile illness using these data found that the blood culture test results were not strongly associated with differential diagnosis,^32^ hence our focus on symptomatology.

## Conclusion

Improving our understanding of the aetiology of acute febrile illness in sub-Saharan Africa remains an important issue for improving diagnosis, patient care, and proper prescription of medications. Bacterial zoonoses, and other common infections transmitted by household animals, transmit many pathogens that cause human fever and may contribute to paediatric morbidity from undifferentiated acute febrile illness.

Future studies should assess the nature of animal contact as a risk factor for fever, the socio-environmental factors that increase pathogen transmission risk from animals, and the types of diagnostic tests that may help identify and treat these infections in a clinical setting.

## Figures and Tables

**Table S1:** Descriptive characteristics of select patient and household characteristics with significant bivariate associ-ations with measured and self-reported fever, using alternate case inclusion criteria of cases without a clinical diagno-sis of malaria (n = 593), rather than a parasite-negative test result

	Measured		Self-Reported	
	Febrile	Afebrile	Febrile	Afebrile
**Overall Rate**	320 (54%)	273 (46%)	349 (59%)	244 (41%)
**Age (years)**	M = 3.29 (SD = 2.64)***	M = 5.24 (SD = 4.21)	M = 3.24 (SD = 2.58)***	M = 5.55 (SD = 4.31)
**Gender**				
**Female**	151 (47%)	143 (52%)	166 (48%)	128 (52%)
**Male**	169 (53%)	130 (48%)	183 (52%)	116 (48%)
**Animal Contact**				
**Yes**	88 (28%)	75 (27%)	93 (27%)	70 (29%)
**No**	232 (72%)	198 (73%)	256 (73%)	174 (71%)
**Type of Animal**				
**Cat**	51 (16%)	46 (17%)	54 (15%)	43 (18%)
**Dog**	35 (11%)	28 (10%)	37 (11%)	26 (11%)
**Sheep/Goat**	1 (<1%)	1 (<1%)	1 (<1%)	1 (<1%)
**Unknown**	1 (<1%)	0 (0%)	1 (<1%)	0 (0%)
**Parent Education**				
**None**	24 (8%)	21 (8%)	24 (7%)	21 (9%)
**Primary**	119 (37%)	99 (36%)	130 (37%)	88 (36%)
**Secondary**	103 (32%)	91 (33%)	114 (33%)	80 (33%)
**Tertiary**	74 (23%)	62 (22%)	81 (23%)	55 (23%)
**Parent Occupation**				
**Professional/skilled labour**	80 (25%)**	99 (36%)	92 (26%)***	87 (36%)
**Trader/unskilled labour/unemployed**	240 (75%)	174 (64%)	257 (74%)	157 (64%)
**Ethnicity**				
**Ga Adangbe**	124 (39%)	89 (33%)	132 (38%)	80 (33%)
**Akan**	103 (32%)	99 (36%)	115 (33%)	87 (36%)
**Ewe**	63 (20%)	66 (24%)	71 (20%)	58 (24%)
**Northern**	23 (7%)	15 (5%)	24 (7%)	15 (6%)
**Unknown**	7 (2%)	4 (1%)	7 (2%)	4 (2%)
**Drug for current illness**				
**None**	88 (28%)***	131 (48%)	97 (28%)***	122 (50%)
**Analgesic**	143 (45%)	101 (37%)	160 (46%)	84 (34%)
**Other**	89 (27%)	41 (15%)	92 (26%)	38 (16%)
**Owns car, motorbike or refrigerator** †				
**Yes**	11 (3%)	0 (0%)	11 (3%)	0 (0%)
**No**	308 (96%)	272 (100%)	336 (96%)	244 (100%)
**Water Source** †				
**Pipe borne water**	10 (3%)	1 (<1%)	10 (3%)	1 (<1%)
**Sachet water**	310 (97%)	272 (99%)	339 (97%)	243 (99%)

* *P* < .05

** *P* < .01

*** *P* < .001

† Associations not measured due to lack of variation

M = mean; SD = standard deviation
